# Electro-Acupuncture Affects the Activity of the Hypothalamic-Pituitary-Ovary Axis in Female Rats

**DOI:** 10.3389/fphys.2019.00466

**Published:** 2019-04-24

**Authors:** Hongmei Zhu, Sha Nan, Chuanguang Suo, Qiulin Zhang, Manli Hu, Rong Chen, Juan Wan, Meng Li, Jianguo Chen, Mingxing Ding

**Affiliations:** College of Veterinary Medicine, Huazhong Agricultural University, Wuhan, China

**Keywords:** hypothalamic-pituitary-ovary axis, electro-acupuncture, hormones, physiological rats, acupoints

## Abstract

Hypothalamic-pituitary-ovary (HPO) axis is a dominant system controlling ovulation during puberty. Electro-acupuncture (EA) has been widely used to cure the reproductive diseases associated with endocrinological disorders. However, whether EA treatment affects HPO axis activity of physiological animals and induces alterations on the hormones in the HPO axis was also unclear. Here, we performed the EA stimuli on bilateral acupoints of Sanyinjiao (SP6) and Zusanli (ST36) on female virgin rats every 3 days and for a total of 5 times. The results showed that GnRH levels in hypothalamus were greatly upregulated in EA-treated rats than untreated animals at day 7 and 13. The serum levels for FSH and LH were severely reduced after EA treatment compared with EA-untreated animals at day 1, while they were greatly increased at day 7 and 13. The serum concentrations of 17β-estradiol were lower in EA-treated rats versus untreated animals at day 7, while they were higher in EA-treated rats than other groups at day 13. However, the progesterone concentrations were lower in EA-treated rats than Control and Sham-EA rats both at day 7 and 13. More importantly, we found that the prostaglandin E_2_ level in serum was reduced in EA-treated rats versus untreated rats at day 1, while they were upregulated at day 7 and 13. Conversely, the norepinephrine level in serum was increased at day 1, while they were decreased greatly in EA-treated rats versus untreated rats at day 7 and 13. The current results demonstrated that EA could modulate homeostasis of HPO axis in physiologic rats, which would be useful to clarify the mechanisms of EA application on pathological and physiological animals or human.

## Introduction

The hypothalamic-pituitary-ovary (HPO) axis plays an important role in female estrous cycle and reproduction (Harris and Santoro, 2011; Clarke, 2014). The hypothalamus exhibits pulsatile release of gonadotrophin releasing hormone (GnRH) into the pituitary, which lead to a similar releasing pattern of follicle stimulating hormone (FSH) and luteinizing hormone (LH) to peripheral blood (Schally, 1970; Schally et al., 1971; Stamatiades et al., 2019). FSH and LH then co-activate the ovarian secretion of 17β-estradiol (E_2_) and progesterone (P_4_) *via* their receptors in the granulosa cells or luteal cells (Albritton, 1941; Nalbandov and Card, 1946; Liu et al., 2018), while the secretion and release of GnRH to the pituitary portal system can be induced and controlled through stimuli received from other mediators in different regions of cerebrum. Diverse mediators such as central neurotransmitters and neuropeptides are integrated in the hypothalamus to regulate the reproductive system (Moore et al., 2018; Saedi et al., 2018). Neurotransmitters of norepinephrine (NE) and prostaglandin E_2_ (PGE_2_), which regulated the activity of neurons in hypothalamus, are potential stimulators affecting the hypothalamic GnRH release (Zwain et al., 2002; Sharif et al., 2013; Fujioka et al., 2017).

On the other hand, acupuncture is a traditional Chinese medical approach to treat patients through stimulating certain acupoints of the body and regulating the flow of Qi and blood in the meridian. Electro-acupuncture (EA) is a modern version of acupuncture that replaces the manual controlling of the needle with electric current (Seo et al., 2017). For decades, EA has been widely used for treating varieties of reproductive diseases due to ovulation and endometrial receptivity disorders, such as infertility, premature incipient ovarian failure, irregular menstruation, polycystic ovary syndrome, and unsuccessful in-vitro fertilization (Stener-Victorin et al., 1996, 2008; Wang et al., 2016). Numerous studies on different acupoints have also been performed to explore the mechanisms of EA treatment. Chen reported that EA at the acupoints of Guanyuan (RN4), Zhongji (RN3), Sanyinjiao (SP6) and bilateral Zigong (EXCA1) influenced the levels of FSH, LH, and E_2_ in plasma and normalized the dysfunctional HPO axis so as to promote ovulation (Chen, 1997). Pasetore et al. reported that acupuncture could improve the LH to FSH ratio in women with polycysticovary syndrome. In rodents, EA at RN4 or SP6 directly regulated the levels of E_2_, FSH, LH, and GnRH in female SAMP8 mice (Wang et al., 2017). Acupuncture at Zusanli (ST36) is able to reverse the spleen deficiency syndrome-induced decrease of testosterone and E_2_ in adult male wistar rats (Wang et al., 2011). Furthermore, EA at ST36 and SP6 acupoints in patients with etomidate anesthesia can mitigate the adrenal cortical inhibition induced by etomidate and can reduce the secretion of catecholamines during surgery (Yu et al., 2014). From the above studies, we can see that the ST36 and SP6 are two common acupoints for EA treatment. However, whether EA affects the physiological animals and what alterations for endocrines of HPO axis would occur after EA treatment on these two acupoints are still unclear.

In the present study, we performed EA on bilateral SP6 and ST36 acupoints on female rats every 3 days and for a total of 5 times. The activity of the HPO axis was evaluated *via* detection of GnRH, FSH, LH, E_2,_ and P_4_ levels in hypothalamus or serum, which could reflect the releasing levels of these hormones at day 1, 7, and 13. In order to further investigate the upstream regulatory factors for GnRH secretion or release, the serum levels of PGE_2_ and NE were also analyzed. The current study on physiology rats would provide a strong foundation and a complementary understanding for EA application in both physiologic and pathologic research.

## Materials and Methods

### Animals

Female Sprague-Dawley rats weighing 250 ± 20 g and aged at 80 ± 5 days were provided by Hubei Provincial Center for Laboratory Animal Research (No.42000600021742). The rats were maintained on a 12-h light:12-h dark regimen, 22 ± 2°C, and 50–70% relative humidity. One week was allowed for their adaption to the surrounding environment. Animals presenting a regular estrous cycle and a same estrous stage detected through vaginal smears during an estrous cycle were housed six per cage with food pellets and water ad libitum. All experimental procedures were approved by the Animal Care and Use Committee of Huazhong Agricultural University.

One hundred and eight rats were randomly divided into three groups: Control (rats were placed in the cylinder without any stimulation), Sham-EA (rats were placed in the cylinder, and needles were inserted into non-acupuncture points and were attached to an acupuncture machine), EA (rats were placed in the cylinder, and needles were inserted into bilateral acupoints of SP6 and ST36 acupoints and were attached to an acupuncture machine). All rats were gently placed into a specially designed polyethylene holder at a fixed time of a day (9:00 a.m.–9:30 a.m.) and for a total of 3 days before the experiment to prevent the animals’ stress response during the experiment. Before the experiment, the estrous stages of the animals were detected *via* the vaginal smears to ensure that the estrous stages of the rats in three groups were consistent. We have also detected that the estrous stages on day 1 (estrous), 4 (proestrous), 7 (proestrous), 10 (diestrous), and 13 (metestrous) of the experiment.

### EA Application

**Figure 1:**
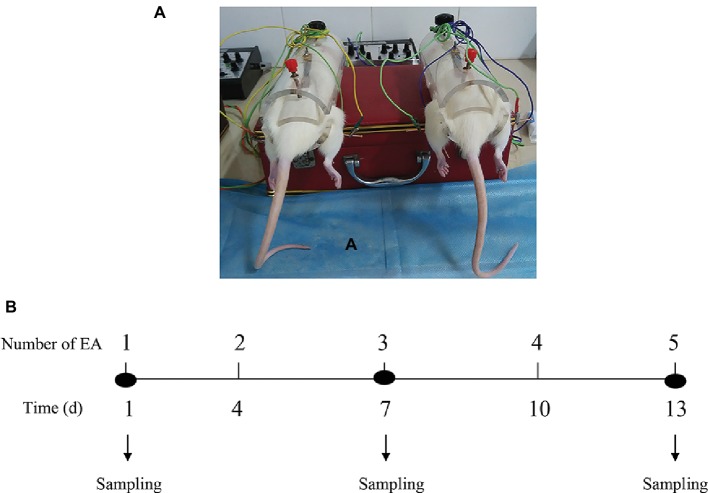
The schematic structure for EA application. **(A)** The diagrams show the dorsal view of the “Zusanli” and “Sanyinjiao” acupoints we used and the electroacupunture procedures in female rats. **(B)** The EA treatment was performed on female rats at day 1, 4, 7, 10, and 13. The tissues were acquired at day 1, 7, and 13 after 30 min of each EA stimuli.

EA stimulations on bilateral SP6 and ST36 acupoints were conducted at a fixed time of a day (9:00 a.m.–9:30 a.m.) based on the previous reports (Kwon et al., 2001; Cabyoglu et al., 2006; Cui et al., 2017). Briefly, each rat was gently placed into a specially designed polyethylene holder with their hind legs and tail exposed. As shown in Figure 1A, stainless-steel needles (0.30 mm in diameter, 13 mm in length) were inserted into bilateral acupoints of ST36 (4 mm lateral to the anterior tuber point of the tibia, which is marked by a notch, 6–7 mm depth) and SP6 (3 mm proximal to the medial malleolus, which is at the posterior border of the tibia, 4–5 mm in depth). Rats were administrated with electrical impulses for 30 min *via* WQ-6F Electronic Acupunctoscope (Xindonghua Electronic Instrument Co., Ltd., Beijing, China). The stimuli parameters were set as 2/100 Hz in frequency (dense-and-disperse mode) and 2 mA in amplitude. The rats in EA group were stimulated at the bilateral acupoints simultaneously. Rats in Control group were assigned as physiological controls and placed in the polyethylene holder for 30 min. Rats in Sham-EA group were treated on four nonacupuncture points (two points were 1–1.5 cm posterior to the bilateral ST36 acupoints, on the posterior aspect of the hind legs with a insertion depth of 6 cm. The other two points were 1–1.5 cm above the SP6 acupoints, on the rear medial aspect of the hind legs with a insertion depth of 4 mm), which were considered as the corresponding Sham points to ST36 or SP6 acupoints and then given the same current as EA group for 30 min according to a previous report (Yu et al., 2014). The first day for EA treatment was recorded as day 1, and the EA was applied at day 1, 4, 7, 10, and 13 (Figure 1B). Rats in each group were kept in the holder without anesthesia during experiment. The samples of blood and brain from 12 rats in each group were collected from the anesthetized rats (pentobarbital, 40 mg/kg) after 30 min of EA stimulation at day 1, 7, and 13 for hormones detection (12 rats/group/sampling).

### qRT-PCR

The hypothalamus was separated from six sacrificed rats in each group after 30 min of EA as previously described (Cui et al., 2017). Briefly, two cuts were made transsectionally at the apex of the optic chiasm and the rostral margin of the mammillary bodies, respectively, on the ventral surface of the brain; the other two cuts were placed sagittally on either edge of the mammillary bodies after the middle slab was placed flat; the last cut was placed coronally just ventrally to the third ventricle. Then, the hypothalamus was isolated and was ground in liquid nitrogen immediately. Half of the hypothalamus was used to RNA analysis (the others were used for hormone detection). Total RNA was extracted using Trizol reagent (Invitrogen, Carlsbad, CA, USA) and was reverse transcripted using a First strand cDNA Synthesis Kit (TOYOBO, Osaka, Japan). The primers (Forward: 5′-GGAGGATCAAATGGCAGAACC-3′ and Reverse: 5′-GAAATGCGGAAGCCCACACAA-3′) were applied for detecting GnRH mRNA expression. Rat GAPDH was used as an internal control. The mRNA of GnRH relative to GAPDH levels was quantified with the 2^−ΔCt^ method, where ΔCt = Ct_target gene_ − Ct_GAPDH_.

### Immunofluorescence and Histology

The brains from the other six sacrificed rats were fixed with 4% paraformaldehyde for 72 h at room temperature. Cutting the brain sections on a cryostat made the last two cuts to isolate the sections comprised of the hypothalamic median eminence (ME). The sections were then embedded in paraffin and sectioned at 5 μm. Three repeated sections were processed in xylene and ethanol for removing the paraffin on the slides. Samples were boiled in sodium citrate buffer (10 mM, pH 6.0) for antigens retrieving for 10 min and were then washed with PBS containing 0.2% Tween-20 (PBST) for three times, 5 min for each time. Then, the samples were blocked with 5% BSA (Sigma) at 37°C for 50 min. After incubated with primary rabbit anti-GnRH antibody (1:100; Bioss; bs10369R) diluted in blocking solution at 4°C overnight, the samples were then washed in PBST for three times, 5 min for each time. An incubation of the samples with the secondary Alexa-Fluor-488-conjugated goat anti-rabbit IgG (H + L) (1:500; protintech; SA00006-2) antibody was done for 1 h at room temperature. After washed with PBST for three times, the nuclei were stained with DAPI (1:2000; Molecular Probes) for 5 min at room temperature. Finally, 50% glycerin was used for sealing. Immunofluorescence images were acquired using the EVOS FL Auto imaging system equipped with an external argon laser. The results were analyzed through the integrated optical density (IOD) by the image pro plus software.

For ovary histology, we separated the ovaries from the other tissues and fixed them in 4% paraformaldehyde for 72 h at room temperature, dehydrated them through graded ethanol series and embedded them in paraffin. The sections were cut at 10 μm and stained with hematoxylin and eosin as described (Parkash et al., 2015).

### Hormone Assays

The blood samples derived from six rats in each group were incubated for 1 h at room temperature. Then, they were centrifuged at 3000 rpm/min, 4°C for 20 min to precipitate the serum. The GnRH in hypothalamus and the serum FSH and LH were detected by Rat GnRH ELISA kit, Rat FSH ELISA kit, and Rat LH ELISA kit, respectively (Shanghai huzhen biology CO., LTD, Shanghai, China). The serum E_2_ and P_4_ levels were analyzed *via* radioimmunoassay (RIA) and were performed as described in the kit manuals (Northern Institute of Biology, Beijing, China). PGE_2_ and NE levels in serum (derived from 6 rats from each group) were all detected according to the Rat PGE_2_ ELISA kit and Rat NE ELISA kit (Shanghai huzhen biology CO). The detection limits of the assay were 5 ng/L for GnRH, 2.344 mIU/ml for FSH, 1.563 mIU/ml for LH, 56.5 pg/ml for E_2,_ 0.2 ng/ml for P_4_, 31.25 pg/ml for PGE_2_, and 1.563 ng/ml for NE. Intra- and inter-assays variations were <10% and <12% for GnRH, were <8% and <10%, respectively for FSH, LH, PGE_2_, and NE, and were <10% and <15% for E_2_ and P_4_. The optical density (OD) for ELISA reaction was read at A450 nm by a microplate reader (BioTek, Winooski, VT, USA). All the samples for the ELISA and the RIA detections were run in triplicate across one assay.

### Statistical Analysis

All data were presented as means ± SD. The statistical analysis was carried out using SPSS 17.0 statistical analysis software. Statistical significance was determined using one-way ANOVA with LSD. A value of *p* < 0.05 was considered significant.

## Results

### EA Stimulation Increased GnRH Expression in Hypothalamus

**Figure 2:**
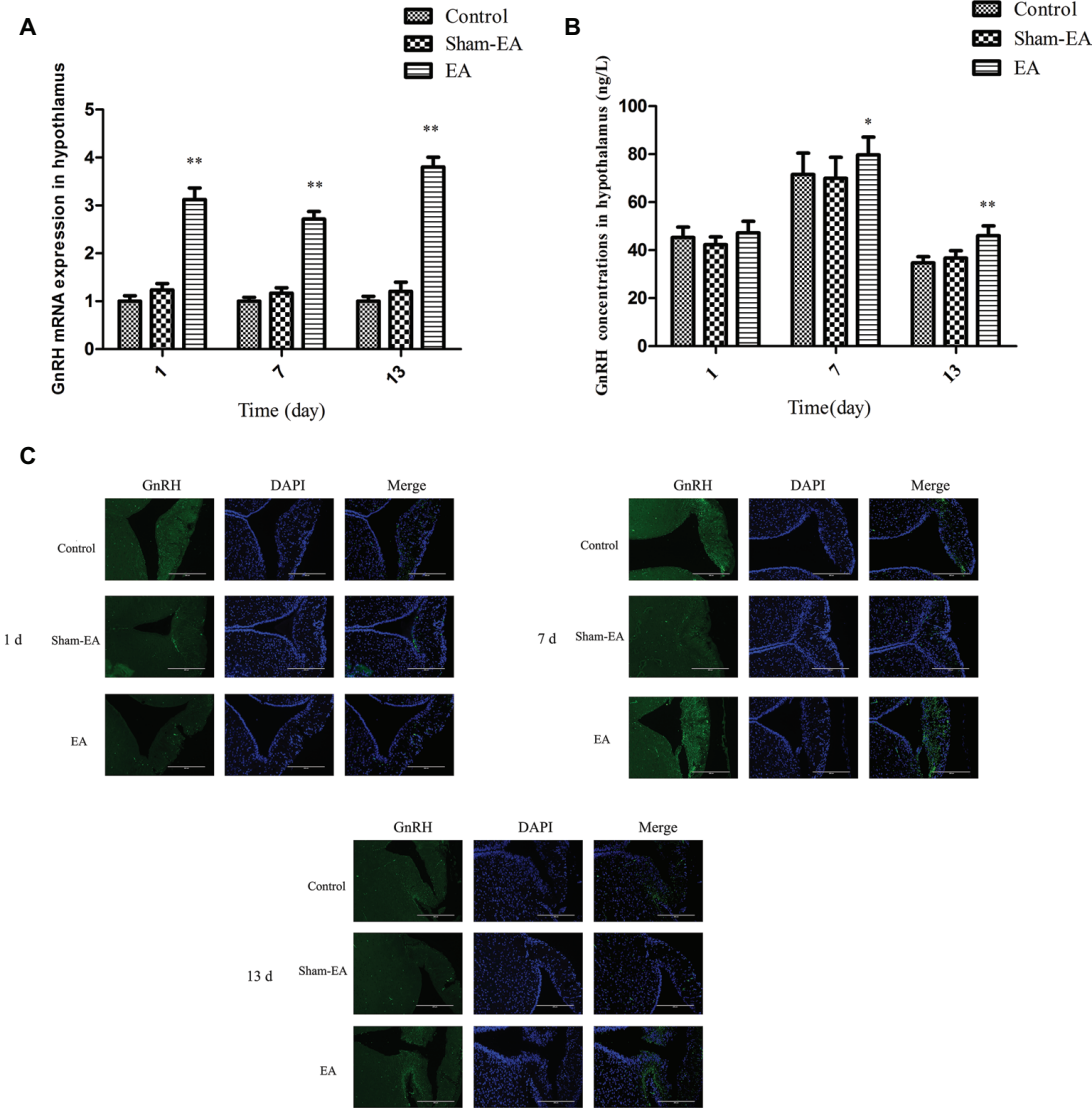
EA simulation facilitated GnRH expression in hypothalamus in female rats. **(A)** The GnRH mRNA level in hypothalamus. **(B)** GnRH concentrations in hypothalamus. **(C)** Dual immunofluorescence showing the expression of GnRH (green) and the neuronal nuclei (blue) in ME in hypothalamus. Scale bars represent 200 μm. Data are presented as means ± SD, ^*^ indicates the change of the GnRH levels are different between EA-treated rats and Control rats, ^*^p < 0.05, ^**^p < 0.01.

The GnRH mRNA and protein expression in hypothalamus were measured at day 1, 7, and 13 after EA stimulation. As shown in Figure 2A, compared with the control and Sham-EA groups, GnRH mRNA expression increased greatly at day 1, 7, and 13 after EA at bilateral SP6 and ST36 acupoints (*p* < 0.01). However, there was no difference in GnRH mRNA in hypothalamus between the Control and Sham-EA groups throughout the study. While, in comparison with the Control and Sham-EA groups, the hypothalamic GnRH content was increased greatly at day 7 and 13 (*p* < 0.05 and *p* < 0.01, respectively) (Figure 2B). Correspondingly, the immunofluorescence for GnRH protein expression in median eminence exhibited a relatively higher positive reaction in EA group than that in the Control and Sham-EA groups at day 7 and 13 analyzed through the IOD (*p* < 0.05, 7 days: EA 3289266.66 ± 955.64 vs Control 267,868 ± 1,376 vs Sham-EA 243755 ± 1,056; *p* < 0.05, 13 days: EA 296685 ± 1,205 vs Control 106,142 ± 1,153 vs Sham-EA 110049 ± 982), while there was no difference in GnRH positive reaction among three groups at day 1 or between the control and Sham-EA groups throughout the study (Figure 2C).

### EA Stimulation Altered FSH and LH Levels in Serum

**Figure 3:**
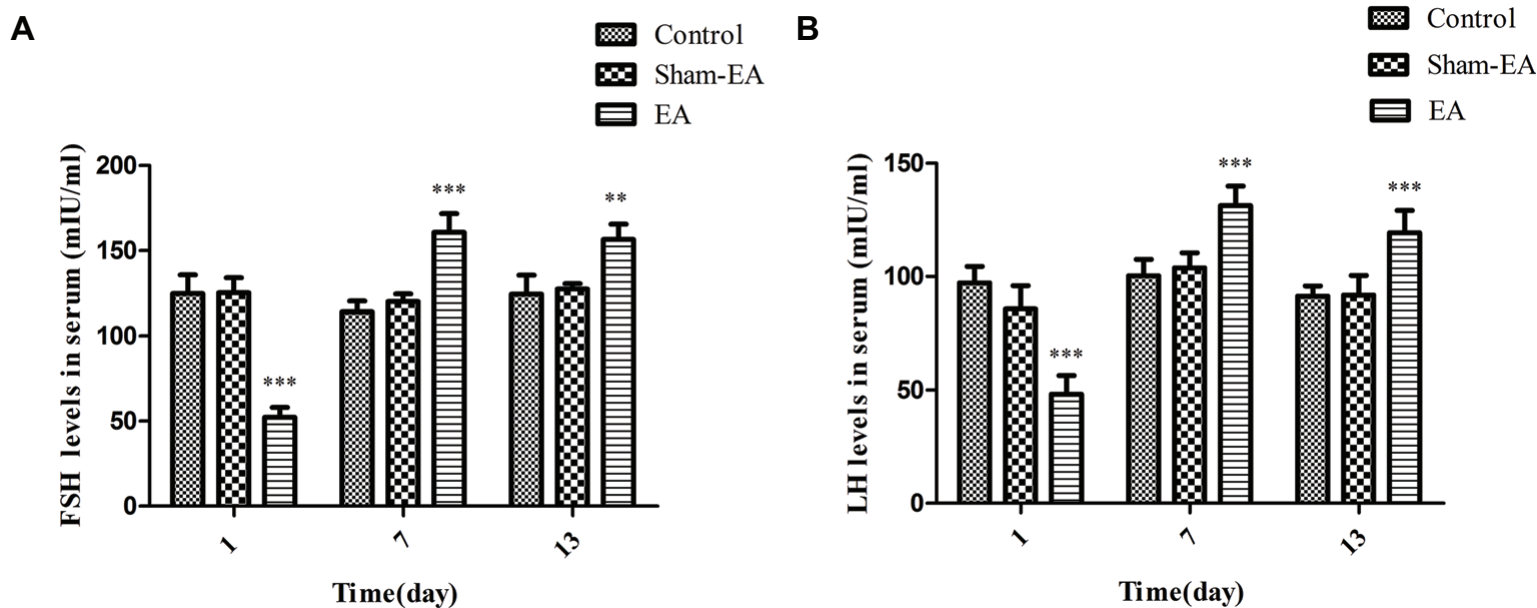
Serum FSH and LH levels at day 1, 7, and 13 after EA treatment. **(A)** Serum FSH levels. **(B)** Serum LH levels. Data are presented as means ± SD, ^*^ indicates the changes of the hormone levels are different between EA-treated rats and the Control or Sham-EA rats, ^**^p < 0.01, ^***^p < 0.001.

The serum concentrations of FSH and LH were determined for evaluating the pituitary function. As shown in Figure 3A, the FSH levels were significantly reduced after EA treatment at day 1 (*p* < 0.01), while they were increased greatly at day 7 and 13 (*p* < 0.001 and *p* < 0.01, respectively) compared with Control and Sham-EA rats. However, there was no difference noted in FSH levels between Control and Sham-EA groups at day 1, 7, and 13.

Serum LH levels showed a similar changes as FSH levels, decreased greatly in EA group at day 1 (*p* < 0.001), but exhibit an significant increase at day 7 and 13 (*p* < 0.001 and *p* < 0.001, respectively) when compared with Control and the Sham-EA groups (Figure 3B), while there was no difference between Control and Sham-EA groups at day 1, 7, and 13.

### EA Stimulation Induced an Different Alterations of E_2_ and P_4_ Levels in Serum

**Figure 4:**
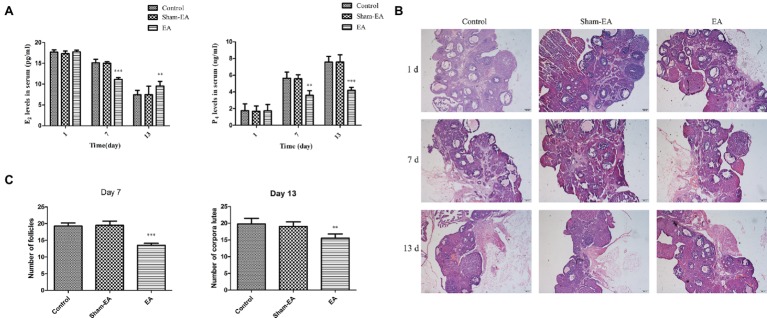
Serum E_2_ and P_4_ levels at day 1, 7, and 13 after EA treatment. **(A)** Serum E_2_ and P_4_ levels. ^*^ indicates the changes of the hormone levels are different between the EA-treated rats and Control or Sham-EA group rats. **(B)** Morphological analysis of ovaries from day 1, 7, and 13. Scale bars represented 200 μm. **(C)** The number of follicles from Control, Sham-EA, and EA group rats at day 7 and the number of corpora lutea in three group rats at day 13. ^*^ indicates the changes of follicles or corpora lutea are different between the EA-treated rats and Control or Sham-EA group rats. Data are presented as means ± SD, ^**^p < 0.01, ^***^p < 0.001.

The serum concentrations of E_2_ and P_4_ were analyzed for evaluating ovary function. As shown in Figure 4A, there was no difference in E_2_ levels among 3 groups at day 1. However, E_2_ levels were severely reduced in EA group at day 7 (*p* < 0.001) and then increased greatly at day 13 (*p* < 0.01) compared with Control and Sham-EA groups. Serum E_2_ concentrations in Sham-EA animals were still not different from the Control rats throughout the study. We found a different changing pattern in P_4_ levels in EA group rats after 5 times of EA treatment at bilateral SP6 and ST36 acupoints from that of Control and Sham-EA groups. This difference was reflected in that P_4_ levels were not different among 3 groups at day 1; thereafter, P_4_ levels were significantly reduced in EA group rats compared with the Control and Sham-EA rats at day 7 and 13 (*p* < 0.01 and *p* < 0.001, respectively) (Figure 4A).

As hormone alterations of E_2_ and P_4_ are responsible for ovary function, we next analyzed whether these alterations would result in ovary histology changes. Indeed, the ovary in EA group exhibited a relative paucity of follicles at day 7 during proestrum and a significant reduction in the number of corpora lutea at day 13 during metestrous compared with Control and Sham-EA groups (Figures 4B,C), indicating the reduced number of ovulations in EA group mice.

### EA Stimulation Induced Contrary Alterations of PGE_2_ and NE Levels in Serum

**Figure 5:**
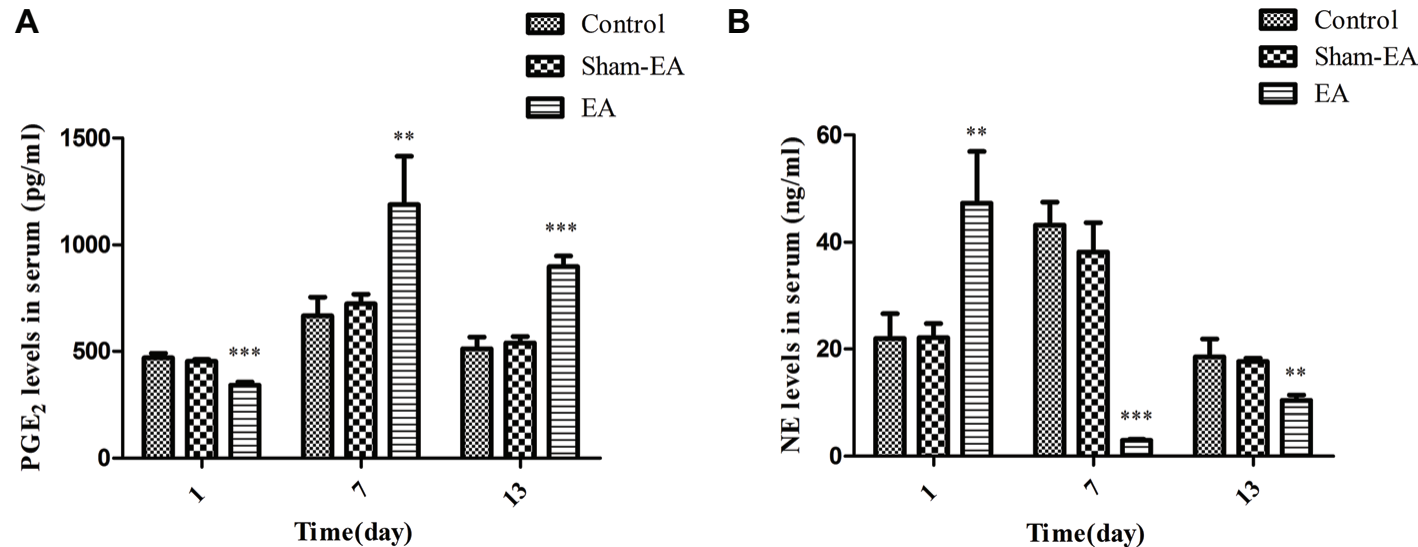
Serum PGE_2_ and NE levels at day 1, 7, and 13 after EA treatment. **(A)** PGE_2_ levels in serum. **(B)** NE levels in serum. Data are presented as means ± SD. ^*^ indicates the changes of the hormone levels are different between the EA-treated rats and the Control or Sham-EA rats, ^**^p < 0.01, ^***^p < 0.001.

Since PGE_2_ and NE are two main regulators for GnRH release and their level-changes would inevitably lead to the alterations of hormones on the HPO axis, we thus analyzed their levels in serum to investigate whether there is an association between their levels and HPO axis activity after EA stimuli. Figure 5A illustrated that PGE_2_ levels were significantly decreased in EA group rats at day 1 (*p* < 0.001) compared with Control and Sham-EA rats. Thereafter, they were greatly higher than in the other two groups at day 7 and 13 (*p* < 0.01 and *p* < 0.001, respectively).

Figure 5B showed that serum NE levels were elevated significantly after EA at bilateral SP6 and ST36 acupoints at day 1 (*p* < 0.01), while they were decreased severely at day 7 and 13 (*p* < 0.001 and *p* < 0.01, respectively) compared with the Control and Sham-EA groups. There was no difference in serum NE levels between the Control and Sham-EA groups throughout the study. These data showed that the levels of PGE_2_ were negatively correlated with that of NE levels, and that both PGE_2_ and NE levels in serum may be closely associated with that of GnRH expression or release in hypothalamus.

## Discussion

Over the past decades, EA has been widely applied for treating human reproductive disorders or for studies regarding with menstruation, ovulation, implantation, and the onset of labor, while these diseases are closely related with the dysregulation of the HPO axis (Mo et al., 1993; Chen, 1997; Stamou et al., 2015; Xiong et al., 2015). However, there are little published data with regard to the effect of EA on the activity of whole HPO axis in physiological animals or human, and the molecular cues responsible for these dynamic morphological changes have not been elucidated so far. Our findings demonstrated that EA treatment on bilateral SP6 and ST36 acupoints induced hormonal fluctuations of HPO axis in physiological rats. These fluctuations specifically manifested in elevated levels of GnRH in hypothalamus, a sudden decreased and then increased levels of FSH and LH, an delayed alterations of serum E_2_ and P_4_ levels, and the contrary alterations of serum PGE_2_ and NE levels after EA treatment.

Studies have demonstrated that different acupoints possess specific effects; different acupoints would activate corresponding central neural regulatory circuits (Cui et al., 2017; Li et al., 2017). In the present study, we have chosen the SP6 and ST36 acupoints, since these two acupoints have been usually set for studies on endocrine disorder or reproductive syndrome (Chen, 1997; Ho et al., 2009; Gao et al., 2013; Wang et al., 2017). Usually, EA application was performed for 20–30 min, once every 2 or 3 days for 7–30 days in total during clinical therapy or studies on infertility (Manni et al., 2005; Wang et al., 2017). Thus, in current research in order to obtain the EA therapeutic effect, we performed EA treatment for 30 min, once every 3 days and for a total of 13 days. In addition, we have even monitored the estrous cycle of all rats and have selected the rats with a 4-day estrous cycle, which were in a same estrous stage for experiment. However, we still cannot assure a same stage of estrous cycle for each EA treatment for it is difficult to reconcile the EA treatment frequency and duration of estrous cycle. Nevertheless, we have detected the estrous stage before each EA treatment and ensured that the estrous stage of rats in Control, Sham-EA, and EA group were consistent. Besides, the patterns of FSH and LH levels were similar accompanied with the remained low and unchanged concentrations during the proestrous and metestrous in Control and Sham-EA groups in current research that was consistent with a previous classical report associated with 4-day estrous cycles at 9:00–10:00 h during a day (Smith et al., 1975). Similarly, the progesterone concentrations were comparable at metestrous and proestrous during 9:00–10:00 am whether in EA treated or untreated rats, demonstrating the normal estrous cycles after 5 times of EA treatment.

After undergoing the EA stimulation on bilateral SP6 and ST36 acupoints at 2 mA, the EA rats experienced elevated GnRH mRNA levels at day 1, 7, and 13, and protein expression in median eminance or hypothalamus at day 7 and 13. This may indicate that EA increased the biosynthesis or secretion of GnRH in physiological female rats. In line with this result, studies on conscious female rabbits found that GnRH levels in the mediobasal hypothalamus increased immediately and significantly after EA stimulation at acupoints of Guanyuan, Zhongji, bilateral SP6 and Zigong (Yang et al., 1994). Further, responses of FSH and LH to GnRH are associated with the pulse frequency and the release of GnRH. The current findings of the coincident FSH and LH alterations at day 1, 7 and 13 may demonstrate that EA at SP6 and ST36 acupoints induced the sudden reduced release at day 1 and then increased secretion of GnRH at day 7 and 13. Moreover, the ovary and its function is the final target of the HPO axis. From Figure 4, we can see that the E_2_ and P_4_ levels did not change in EA group rats at day 1 but then decreased at day 7 at proestrous, while at last, the E_2_ and P_4_ levels show different alterations at day 13 during metestrous. The results may indicate that the instantaneous EA did not affect ovary function at day 1, while EA-induced prolonged FSH and LH alterations may impaired the follicle development at proestrous and then the corpora lutea formation at metestrous, which result in the increased E_2_ levels and decreased P_4_ levels at day 13. Besides, it has been reported that electrical stimulation of the autonomic nerves to the ovary reduced the secretion rate of estradiol or testosterone from the ovary (Kagitani et al., 2008; Uchida and Kagitani, 2014); EA stimulation on the specific muscles of hindlimb increased the ovarian blood flow or changed some specific genes expression from the ovary (Stener-Victorin et al., 2004; Manni et al., 2005), while acupoints were found with abundant distribution of nerve bundles, motor points of neuromuscular attachments, and blood vessels (Zhou and Benharash, 2014). The mechanism by which acupuncture exerts its function is very complex; it involves neural and humoral regulation (Long et al., 2015). These results implied that the effect of EA at SP6 and ST36 acupoints on the HPO axis or ovary may be a comprehensive regulation of neuro-endocrine system, which contributed to the level changes of E_2_ and P_4_.

GnRH is the primary brain signal in HPO axis, which is responsible for release of FSH, LH, or even E_2_, and P_4_. Numerous neurotransmitters and neuropeptides such as dopamine, norepinephrine, γ-aminobutyric acid (GABA), neuropeptide Y, neurotensin, β-endorphin, and glutamate could alter GnRH neuronal activity through direct or indirect actions on hypothalamus (Smith and Jennes, 2001; Han and Herbison, 2008; Tobari et al., 2014). Also, prostaglandins are implicated as intermediates involved in the hypothalamic-pituitary axis and that PGE_2_ promotes gonadotropin secretion through regulating the activity of neurons that release GnRH in the hypothalamus in ovariectomized rats (Harms et al., 1973; Naidich et al., 2010; Clasadonte et al., 2011; Fujioka et al., 2017). In current research, the significant and consistent alterations of basal PGE_2_ concentrations to FSH and LH levels in serum at day 1, 7, and 13 (Figures 3 and 5A), respectively, suggested that PGE_2_ participated in EA-induced GnRH and then subsequent FSH or LH activation, while the significant changes of NE concentrations in serum, which was contrary to the PGE_2_, FSH, and LH levels after EA treatment at day 1, 7, and 13, indicated that serum NE may exert an inhibitory effect on GnRH release after EA at SP6 and ST36 acupoints. In addition, the regulation for HPO axis is very complex, the ovarian steroid hormone would feed back at both the hypothalamus and the anterior pituitary, and it is also difficult to exclude the possibility that EA increased brain β-endorphin or other chemicals to disturb the release or pulse frequency of the GnRH, which contributed to the up-regulation or down-regulation of FSH or LH.

Nevertheless, the GnRH, FSH, or LH levels in current study were contrary to some previous reports that these hormones were reduced in ovariectomized or pathological rats after EA for 20 min, once every other day for 15 times, or once a day for 28 times (Cheng and Tian, 2012; Jiang et al., 2017; Wang et al., 2017). This discrepancy may be caused by different methods (acupoints and times, duration, and intervals of EA) and different animal models (physiological vs ovariectomized or pathological) used among several studies. All together, these data uncover a hiterto unknown physiological role for EA treatment at bilateral SP6 and ST36 acupoints in the control of the hormone secretion on HPO axis. Since the HPO axis is regulated by numerous mechanisms *in vivo* and it is difficult to estimate the combined response to EA treatment on the pathology animals models, the key advantages in current research offered the comprehensive understanding of HPO response to EA stimulation and pave the way for the development of EA treatment strategies in the disorder of HPO axis. In addition, they also enlighten the role of EA treatment on physiological animals or human, which would disturb the HPO homeostatis and impaired the ovary function.

## Author Contributions

MD designed the experiments. HZ and QZ performed acupuncture. HZ, CS, RC and ML performed ELISA and RIA analysis. JC and HZ performed qPCR. SN performed the immunofluorescence. HZ, MH and JW performed the data analysis. HZ wrote the manuscript.

### Conflict of Interest Statement

The authors declare that the research was conducted in the absence of any commercial or financial relationships that could be construed as a potential conflict of interest.
